# MAS-YOLOv11: An Improved Underwater Object Detection Algorithm Based on YOLOv11

**DOI:** 10.3390/s25113433

**Published:** 2025-05-29

**Authors:** Yang Luo, Aiping Wu, Qingqing Fu

**Affiliations:** 1 School of Electronic Information and Electrical Engineering, Yangtze University, Jingzhou 434023, China; 2023750177@yangtzeu.edu.cn; 2 School of Computing Science and Artificial Intelligent, Suzhou City University, Suzhou 215104, China; apwu@szcu.edu.cn

**Keywords:** underwater object detection, YOLOv11, attention mechanism, detection head, loss function, multi-scale feature learning

## Abstract

To address the challenges of underwater target detection, including complex background interference, light attenuation, severe occlusion, and overlap between targets, as well as the wide-scale variation in objects, we propose MAS-YOLOv11, an improved model integrating three key enhancements: First, we introduce the C2PSA_MSDA module, which integrates multi-scale dilated attention (MSDA) into the C2PSA module of the backbone, enhancing multi-scale feature representation via dilated convolutions and cross-scale attention. Second, an adaptive spatial feature fusion detection head (ASFFHead) replaces the original head. By employing learnable spatial weighting parameters, ASFFHead adaptively fuses features across different scales, significantly improving the robustness of multi-scale object detection. Third, we introduce a Slide Loss function with dynamic sample weighting to enhance hard sample learning. By mapping the loss weights nonlinearly to detection confidence, this mechanism effectively enhances the overall detection accuracy. The experimental results demonstrate that the improved model yields significant performance advancements on the DUO dataset: the recall rate is enhanced by 3.7%, the F1-score is elevated by 3%, and the mAP@50 and mAP@50-95 attain values of 77.4% and 55.1%, respectively, representing increases of 3.5% and 3.3% compared to the baseline model. Furthermore, the model achieves an mAP@50 of 76% on the RUOD dataset, which further corroborates its cross-domain generalization capability.

## 1. Introduction

With the surging global demand for marine resources, China has incorporated the strategy of becoming a maritime power into the core of its national development and strengthened marine protection [1]. However, the superposition of human activities and climate change leads to significant ecological degradation in the coastal waters, especially the decline of fishery resources, ecological fragmentation, and the sharp decline of biodiversity [2]. Therefore, our country is constructing an oceanic ranch for the systemic recovery of fishery resources and marine ecological function [3].

Underwater imaging primarily relies on sonar sensors and optical cameras. Sonar enables long-range perception but has low resolution [4,5], while optical cameras provide high-resolution imaging suited for the close-range detection of small marine organisms. However, underwater environments degrade image quality through light scattering, refraction, and absorption, causing color distortion and detail loss [6], which hinder object detection performance.

Existing object detection algorithms are generally categorized as traditional machine learning or deep learning approaches [7]. In underwater applications, conventional methods follow a three-stage pipeline: region proposal generation through multi-scale sliding windows, feature extraction with descriptors (e.g., SIFT, HOG), and classification using models like SVM and AdaBoost. The detection results are subsequently refined through post-processing techniques such as Non-Maximum Suppression (NMS).

Traditional underwater target detection methods have made limited progress in recent years. Cheng et al. [8] enhanced texture features using gray-level co-occurrence matrices but required intensive computation, rendering them ill-suited for real-time applications. Yahya et al. [9] improved spatial alignment via bounding-box division frameworks yet maintained limited robustness against dynamic lighting and particle interference. Susanto et al. [10] reported a 37% accuracy drop in turbid waters for color-based features, underscoring the limits of relying on single features. These approaches remain dependent on handcrafted features, primarily constrained by poor environmental adaptability and low computational efficiency in complex underwater settings.

In recent years, deep learning-based object detection algorithms have outperformed traditional methods due to their ability to automatically extract hierarchical features. Object detection algorithms based on deep learning can be divided into single-stage and dual-stage object detection. The single-stage detectors adopt an end-to-end regression strategy to directly perform target classification and bounding-box regression on the feature map, and the representative algorithms include YOLO [11] series and SSD [12]. The two-stage detectors adopt a cascading structure, first generating region proposals, and then implementing refined classification and coordinate regression, including R-CNN [13], Faster R-CNN [14], MR-CNN [15], FPN [16], etc., which are usually computationally complex.

Deep learning has advanced object detection, yet underwater environments pose persistent challenges: light scattering and absorption degrade the image quality, dynamic particles induce blurring, and small, low-contrast targets prove difficult to discern. Additionally, limited computational resources restrict real-time algorithm deployment. In response to these challenges, researchers have proposed a series of improvement methods from multiple dimensions:

In terms of small object detection, Xin et al. [17] used a hybrid dilated CNN to replace the VGG16 backbone network in Faster R-CNN, which effectively alleviated the problem of small-target feature loss caused by traditional pooling operations. Ye et al. [18] enhanced multi-scale detection by increasing the prediction scale of YOLOv3-SPP and optimizing the anchor frame distribution in combination with K-Means++.

In view of the degradation of underwater images, Cao Jianrong et al. [19] integrated the Dark Channel Prior algorithm in YOLOv5 preprocessing to compensate for color distortion physically. Liu Ping et al. [20] designed a cGAN-based module that generates high-quality images via adversarial training, enhancing YOLOv3’s detection robustness. Chang et al. [21] developed a 3D position-embedding Transformer that jointly models spatial, color, and contextual features for end-to-end enhancement. Cao et al. [22] proposed BG-YOLO, leveraging parallel enhancement and detection branches with feature steering to improve mAP effectively without increasing the computational load.

At the level of feature extraction optimization, Yan Zhang et al. [23] and Knausgard’s team [24] improved the backbone network through the channel attention mechanism, and the SENet module significantly enhanced the representation ability of high-frequency features by explicitly modeling the dependencies between channels. The Underwater-YCC model proposed by Chen et al. [25] innovatively uses Conv2Former as the neck network, which integrates the local perception advantage of convolution with the global modeling ability of Transformer to effectively process the feature extraction of underwater fuzzy images.

To address model lightweighting, Xin Shiao’s team [26] incorporated the ShuffleNetv2 backbone within YOLOv7, augmented by BiFormer attention and BiFPN, achieving a 43% parameter reduction while preserving accuracy. Liu Xiangju et al. [27] proposed DCN-YOLOv5, employing deformable convolutions for enhanced detection of deformed targets. Zhao et al. [28] developed BGLE-YOLO, reducing the computational cost and model size through multi-scale convolutions and global–local feature fusion.

It is worth noting that the USSTD-YOLOv8n model proposed by Yi et al. [29] uses the Content-Aware Feature Reconstruction Upsampling Operator (CARAFE) to achieve 91.2% mAP in a low-visibility environment on URPC2022 datasets, but its actual deployment effect still needs to be further verified by cross-domain generalization experiments.

Although previous studies have achieved certain successes, underwater object detection still has significant room for improvement in terms of detection accuracy and real-time requirements. YOLOv11 utilizes SPFF and C3K2 modules to extract multi-granular features through hierarchical pyramids, enhanced by dynamic anchor matching to strengthen feature learning for blurred contour targets. While small target detection remains challenging, the improved C2PSA module suppresses water ripple noise through local–global feature interaction, yet it struggles to model the global contextual dependencies of small targets in complex scenes. The detection head’s dual-DWConv design reduces the parameters/computation by 41% while maintaining real-time performance. However, its NMS-free training strategy risks missed detections in occlusion-dense scenarios. Our enhanced framework addresses these limitations through adaptive feature recalibration and occlusion-aware detection mechanisms.

Key improvements include the following: (1) Integrating multi-scale dilated attention (MSDA) [30] into the backbone’s C2PSA module (C2PSA_MSDA) to enhance the modeling ability of targets at different scales. (2) The adaptive feature fusion mechanism [31] (ASFF) was used to construct ASFFHead in the detection head to optimize the fusion effect of multi-scale features. (3) Based on classification loss, a Slide Loss function [32] based on a dynamic weighting strategy is introduced to improve the model’s attention to indistinguishable samples, to enhance the overall detection accuracy.

The rest of this paper is structured as follows: Section 2 introduces related work; Section 3 details the principle of MAS-YOLOv11; Section 4 performs evaluation of MAS-YOLOv11 in combination with the experimental results; Section 5 summarizes the full text.

## 2. Related Work

### 2.1. YOLOv11

YOLOv11 is one of the outstanding target detection algorithms in the YOLO series. It combines precision and speed, and supports parallel multi-task processing. It has a wide range of application prospects in terms of deployment efficiency. Its overall structure includes the input layer, the backbone network, the neck, and the detection head.

The input layer integrates Mosaic-Plus augmentation with adaptive anchor matching, dynamically adjusting intersection-over-union thresholds to optimize positive/negative sample allocation. The backbone’s C3K2 module employs a tri-branch architecture: a 3 × 3 convolutional cascade in the main branch, 1 × 1 channel compression, and 5 × 5 dilated convolution in auxiliary branches. The enhanced C2PSA incorporates selective residual connections to improve gradient flow, coupled with position-sensitive attention (PSA) through multi-head attention and feedforward networks. The BiFPN-optimized neck enables bidirectional cross-scale fusion via cross-layer residual connections, while channel-attention-guided aggregation learns hierarchical feature weights. The decoupled detection head combines depthwise separable convolution with IoU-aware mechanisms, balancing accuracy with computational efficiency.

Through structural optimization and module improvement, YOLOv11 achieves detection accuracy, running speed, and multi-task processing ability, making it suitable for a variety of real-time scenarios. However, its ability to detect small objects is insufficient, such as at long distances or in the event of serious occlusion of aquatic organisms. The stability of pose estimation in dynamic scenes is poor. At the same time, the model is based on a general training dataset, its cross-domain generalization ability is limited, and it is difficult to adapt directly to the complex underwater environment. Therefore, to improve small-target awareness, enhancing the robustness of underwater scenes and adding features of underwater imaging optimization is the key to further enhance YOLOv11’s underwater target direction.

### 2.2. Attention Mechanisms

An attention mechanism simulates human selective cognition through dynamic weight allocation, focuses on key information, and improves feature expression effects [33]. The attention mechanisms in deep learning mainly include four types: Spatial attention locates the target area through spatial transformation, but it is limited by rigid transformation. Channel attention models the channel relationship through the global context, but the spatial details are insufficient. Temporal attention focuses on the temporal dependence of sequence data, which is suitable for video detection tasks. Branch attention enhances the feature selection ability with multi-branch architecture. In view of the heterogeneous characteristics of the underwater environment, this paper focuses on exploring the co-optimization mechanism of spatial and channel attention to improve the feature discrimination power in complex scenes.

A typical architecture for spatial attention mechanisms is Spatial Transformer Networks (STNs) [34], which adapt to the geometric structure of input features through affine transformation operations, maintain feature consistency in deformation scenarios such as rotation and scaling, and significantly enhance the spatial robustness of the model. The classical model of the channel attention mechanism is the Squeeze-and-Excitation Network (SENet) [35]. The global pooling compression feature map is adopted. The nonlinear dependence relationship between channels is modeled at the full connection layer.

## 3. Materials and Methods

Although YOLOv11 is one of the most advanced object detection algorithms and is widely applied across various domains, its performance in underwater target detection remains challenged by sample overlap, blurring, and occlusion due to the unique imaging conditions of underwater environments. These densely packed targets make it difficult for the network to distinguish object boundaries, thereby increasing localization challenges and reducing the detection accuracy.

**Figure 1:**
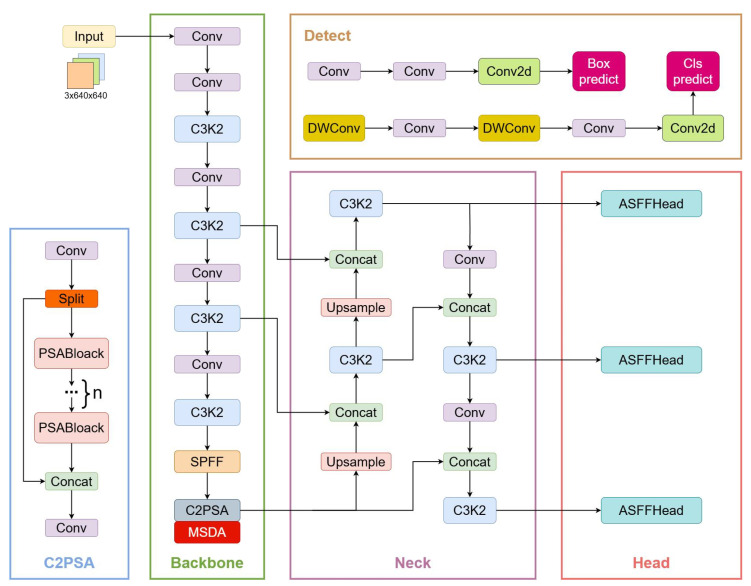
MAS-YOLOv11 model, where the MSDA was embedded in the C2PSA module and the original YOLO v11 detection head was replaced with ASFFHead.

To achieve accurate and efficient underwater target detection, this paper proposes MAS-YOLOv11, an enhanced underwater target detection network based on YOLOv11, as illustrated in Figure 1. The key improvements are as follows:

1. Multi-scale dilated attention mechanism (MSDA) integration: The MSDA module is embedded into the C2PSA module of the YOLOv11 backbone network, forming a new module called C2PSA_MSDA. This module effectively captures both fine-grained local details and broader contextual information. By integrating dilated convolution and self-attention mechanisms, the receptive field is significantly expanded while maintaining computational efficiency. This enhancement improves the network’s ability to capture long-range dependencies, strengthens global contextual understanding, and enhances feature learning efficiency and representational capacity.
2. Adaptive feature fusion detection head (ASFFHead): The original Detect detection head is replaced with ASFFHead, which introduces an adaptive weighting mechanism to optimize multi-scale feature fusion. Traditional multi-scale fusion may suffer from conflicts and inconsistencies between features at different scales. ASFFHead effectively mitigates these issues, ensuring seamless fusion of information across scales. This is particularly beneficial in underwater target detection, where objects exhibit significant scale variations and are often occluded. By dynamically integrating multi-scale features, ASFFHead enhances both detection accuracy and model robustness in complex underwater environments.
3. Slide Loss function with dynamic weighting strategy: To improve the model’s learning capability on difficult samples, the Slide Loss function is incorporated into the training process. Unlike conventional loss functions that assign uniform weights to all samples, Slide Loss dynamically adjusts the loss contribution of each sample based on its difficulty level. This strategy places greater emphasis on hard-to-classify samples, ensuring that the model prioritizes these challenging cases during training. As a result, the optimization process is refined, leading to improved generalization and enhanced feature learning for difficult samples.

### 3.1. C2PSA_MSDA Module

In the YOLOv11 model, the C2PSA module enhances feature extraction and representation by dynamically weighting the feature map through the Pointwise Spatial Attention (PSA) block. This mechanism allows the model to selectively focus on key regions within an image, significantly improving its ability to capture critical details and suppress less informative areas. However, in complex environments such as underwater scenes, objects often exhibit diverse scales, occlusions, and varying visibility conditions, which pose additional challenges to effective feature representation.

**Figure 2:**
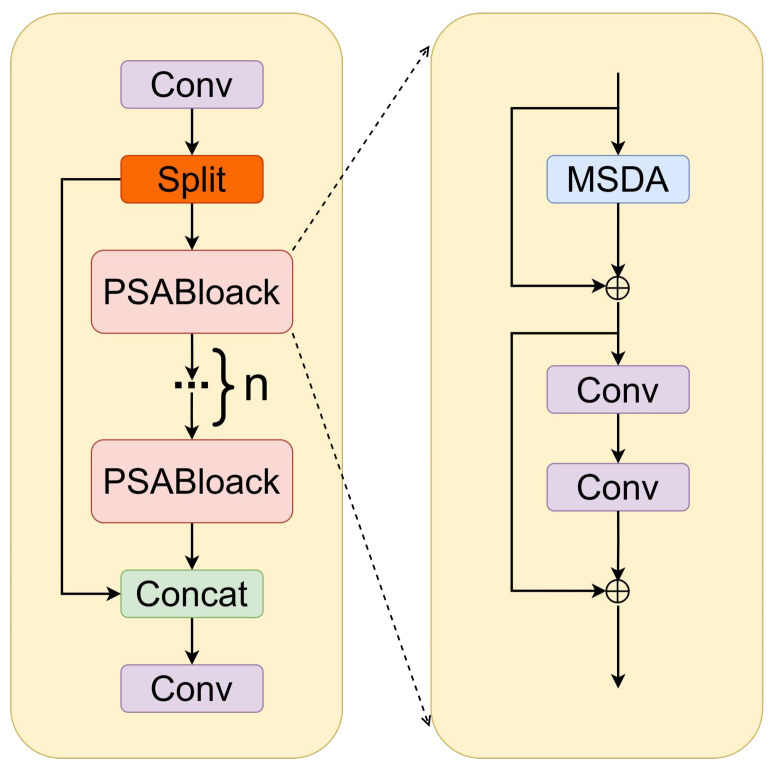
C2PSA_MSDA module.

To address these challenges, as illustrated in Figure 2, this study further integrates the multi-scale dilated attention mechanism (MSDA) into the PSA block, forming the enhanced C2PSA_MSDA module. The MSDA mechanism is designed to improve the network’s ability to extract multi-scale contextual information by introducing dilated convolutions with varying dilation rates. This adaptive approach enables the model to capture fine-grained local features while simultaneously aggregating broader contextual information across different receptive fields. Unlike conventional attention mechanisms that rely on fixed spatial transformations, C2PSA_MSDA adaptively adjusts its receptive field, ensuring that important structural details at different scales are effectively preserved and utilized.

By aggregating multi-scale features, the C2PSA_MSDA module significantly enhances the robustness of feature representations, allowing the network to more effectively detect and differentiate targets across diverse underwater environments. This improvement is particularly crucial in mitigating the adverse effects of blurring, occlusion, and uneven lighting conditions commonly found in underwater imagery. Moreover, the incorporation of multi-scale spatial attention ensures that the model remains sensitive to both fine-scale details and large-scale contextual information, thereby improving both localization precision and classification accuracy. Overall, the integration of C2PSA_MSDA into the YOLOv11 backbone provides a more adaptive, scale-aware, and context-sensitive feature extraction framework, enhancing the model’s ability to process complex underwater scenes with greater accuracy and robustness.

**Figure 3:**
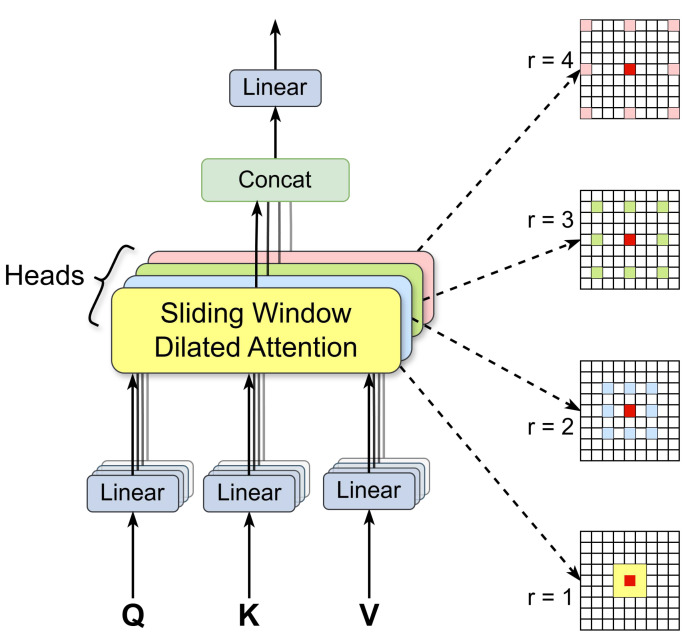
MSDA module, which describes the working principle of MSDA in the C2PSA_MSDA module. First, the feature map channels are split into multiple heads. Each head performs a self-attention operation on a query patch within a colored region centered on a red window, using a distinct dilation rate. The features from different heads are then concatenated and passed through a linear layer. By default, we use four dilation rates: r = 1, 2, 3, and 4, corresponding to receptive field sizes of 3 × 3, 5 × 5, 7 × 7, and 9 × 9, respectively.

The working principle of the multi-scale dilated attention mechanism (MSDA) in the C2PSA_MSDA module is illustrated in Figure 3. The core objective of MSDA is to enhance the model’s ability to capture both fine-grained and high-level contextual features by employing a multi-scale dilated self-attention mechanism, particularly when processing objects and textures at varying spatial scales. The detailed operational process of MSDA is as follows:

1. Feature projection and segmentation: MSDA begins by applying a linear projection to the input feature map, generating the corresponding query (Q), key (K), and value (V) matrices. To enable efficient multi-scale feature extraction, the channel dimension of the feature map is divided into multiple attention heads, where each head processes different feature subspaces independently. This multi-head architecture allows the model to capture a diverse set of image features by learning independent attention patterns across different spatial scales.
2. Multi-scale Sliding Window Dilated Attention (SWDA) operation: Within each attention head, MSDA introduces different dilation rates (r) to perform the multi-scale Sliding Window Dilated Attention (SWDA) operation. The dilation rate determines the size of the receptive field of the sliding window, enabling the model to extract contextual information across different scales. By default, the dilation rates are set as r = {1, 2, 3, 4}, corresponding to 3 × 3, 5 × 5, 7 × 7, and 9 × 9 receptive fields, respectively. The smaller receptive fields focus on local textures and fine object structures, while the larger receptive fields capture broader contextual relationships, ensuring a balance between local feature refinement and global scene understanding. This design is particularly effective in underwater environments, where objects of varying sizes, occlusions, and complex backgrounds pose significant detection challenges.
3. Attention computation and feature fusion: To compute self-attention, each attention head evaluates the similarity between the query (Q) and key (K) to determine the relative importance of different spatial regions. The value (V) matrix is then weighted and aggregated based on these attention scores, allowing the model to dynamically emphasize critical features at multiple scales. Finally, the multi-scale features extracted from different heads are fused through a linear projection layer, refining and integrating the information into a comprehensive feature representation.

By embedding MSDA into the C2PSA module, the model achieves more effective multi-scale feature extraction, significantly expands its receptive field, and enhances its robustness to occlusions and noise. This improvement is particularly crucial for underwater target detection, where objects are often densely clustered, partially obscured, or blurred due to water distortion. The C2PSA_MSDA module allows the model to adaptively focus on key regions across different scales, improving both detection accuracy and robustness in complex underwater environments.

### 3.2. ASFFHead

The adaptive spatial feature fusion (ASFF) mechanism is designed to filter out conflicting information across different scales while retaining essential and complementary features by dynamically learning feature fusion weights at each spatial location. Unlike traditional feature pyramid structures, which may suffer from inconsistencies in multi-scale feature fusion, ASFF enables the network to adaptively select the most informative contributions from different feature scales at each spatial position. This ensures a seamless and balanced feature representation, which is crucial for improving detection accuracy, particularly in challenging environments such as underwater object detection, where scale variations and occlusions are common. To fully leverage this capability, ASFF was integrated into the original Detect module of YOLOv11, forming a new detection head, ASFFHead. By effectively fusing high-level semantic features and low-level fine-grained features, ASFFHead enhances the model’s ability to detect objects of varying sizes, leading to more precise bounding-box localization and classification accuracy.

**Figure 4:**
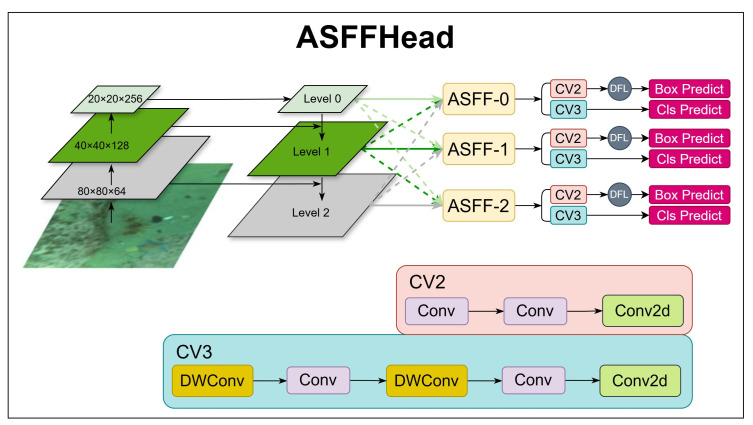
ASFFHead structure describing the ASFFHead workflow.

As illustrated in Figure 4, the neck layer of the model outputs three feature maps of different scales, namely, Level 0, Level 1, and Level 2, each possessing distinct spatial resolutions and levels of abstraction. To facilitate effective fusion while ensuring that all feature maps share the same spatial dimension, feature rescaling operations are applied, including 1/2 downsampling, 1/4 downsampling, and upsampling, depending on the level.

Within ASFFHead, the adaptive fusion process is performed at each level, denoted as ASFF-0, ASFF-1, and ASFF-2, corresponding to the three output levels of the neck layer. Each ASFF module applies a learnable weighted summation across different levels, utilizing the adaptive coefficients α, β, and γ, which dynamically determine the importance of features from each scale. These learnable fusion weights ensure that the model assigns higher importance to the most relevant feature maps at each spatial location, thereby effectively mitigating scale inconsistencies and improving detection robustness. Taking ASFF-2 as an example, at each spatial position (*i*, *j*), the ASFFHead fuses features from multiple levels using the adaptive weights $\alpha_{ij}^{l}$, $\beta_{ij}^{l}$, and $\gamma_{ij}^{l}$, The fusion formula is as follows:

(1) $$F_{ij}^{l}=\alpha_{ij}^{l}\cdot x_{ij}^{0\to l}+\beta_{ij}^{l}\cdot x_{ij}^{1\to l}+\gamma_{ij}^{l}\cdot x_{ij}^{2\to l}$$

where $F_{ij}^{l}$ is the output feature map of level *l* at position (*i*, *j*); $x_{ij}^{0\to l}$ is a feature vector that is rescaled from level *n* to level l at position (*i*, *j*); $\alpha_{ij}^{l}$, $\beta_{ij}^{l}$, and $\gamma_{ij}^{l}$ are adaptive learning weights that meet the following formula; and $\alpha_{ij}^{l},\;\beta_{ij}^{l},\;\gamma_{ij}^{l}\in \left[0,\;1\right]$.

(2) $$\alpha_{ij}^{l}+\beta_{ij}^{l}+\gamma_{ij}^{l}=1$$

(3) $$\alpha_{ij}^{l}=\frac{e^{\lambda_{\alpha_{ij}}^{l}}}{e^{\lambda_{\alpha_{ij}}^{l}}+e^{\lambda_{\beta_{ij}}^{l}}+e^{\lambda_{\gamma_{ij}}^{l}}}$$

(4) $$\beta_{ij}^{l}=\frac{e^{\lambda_{\beta_{ij}}^{l}}}{e^{\lambda_{\alpha_{ij}}^{l}}+e^{\lambda_{\beta_{ij}}^{l}}+e^{\lambda_{\gamma_{ij}}^{l}}}$$

(5) $$\gamma_{ij}^{l}=\frac{e^{\lambda_{\gamma_{ij}}^{l}}}{e^{\lambda_{\alpha_{ij}}^{l}}+e^{\lambda_{\beta_{ij}}^{l}}+e^{\lambda_{\gamma_{ij}}^{l}}}$$

where $\lambda_{\alpha_{ij}}^{l}{,\;\lambda }_{\beta_{ij}}^{l},\;and\;\lambda_{\gamma_{ij}}^{l}$ are control parameters calculated from the rescaled feature map by 1 × 1 convolution.

By dynamically adjusting the fusion weights, ASFF ensures that each spatial position selectively incorporates the most valuable information from multiple scales, leading to a more discriminative feature representation. This not only enhances the spatial precision and semantic richness of the model but also significantly improves the object detection performance, particularly in complex multi-scale scenarios such as underwater environments.

### 3.3. Loss Function

In object detection tasks, the issue of imbalanced sample distribution is a critical factor that constrains model performance. Empirical observations reveal that datasets are often dominated by easily classifiable positive samples with a high intersection-over-union (IoU) ratio, such as objects with clear backgrounds and precise positioning, and simple negative samples with a low IoU, where the prediction boxes are significantly misaligned with the actual target. Conversely, hard samples—those with IoU values near the classification threshold, such as occluded objects, small targets, or predictions with blurred boundaries—are relatively sparse. This long-tailed distribution inherently biases the training process, causing the loss function to be predominantly influenced by simple samples. As a result, the gradient signals from hard samples are overwhelmed during backpropagation, significantly impairing the model’s ability to generalize to complex scenarios, and ultimately degrading both the detection accuracy (mean average precision, mAP) and localization robustness.

To address this challenge, this paper uses the Slide Loss function based on a dynamic weighting strategy to optimize the classification accuracy of the model. Traditional approaches typically rely on fixed thresholds to differentiate positive and negative samples; however, such thresholds fail to adapt to varying dataset distributions. Instead, our method introduces an adaptive threshold *μ*, which is computed as the mean *IoU* of all prediction boxes and their corresponding ground-truth bounding boxes. This threshold enables dynamic sample categorization: samples with *IoU* ≥ *μ* are classified as positive, while those with *IoU* < *μ* are designated as negative. Mathematically, the adaptive threshold is defined as follows:

(6) $$\mu =\frac{1}{N}\sum_{i=1}^{N}{IoU}_{i}$$

where *N* represents the total number of samples, and ${IoU}_{i}$ denotes the *IoU* of the i-th prediction box with its corresponding ground-truth box. This dynamic partitioning strategy not only mitigates manual hyperparameter tuning but also enhances the model’s generalization capabilities.

Building upon this foundation, the Slide Loss function dynamically adjusts sample weights through a piecewise exponential function, which is defined as follows:

(7) $$f\left(x\right)=\left\{\begin{array}{c} 1\;,\;x\leq \mu -0.1,\;\\ {\;e}^{1-\mu }\;,\;\mu -0.1<x<\mu \\ e^{1-x}\;,\;x\geq \mu . \end{array}\right.$$

where *x* represents the *IoU* of a prediction box, and the weight function *f(x)* follows a carefully designed assignment principle.

1. Suppression of simple negative samples (x≤μ−0.1): A fixed weight of 1 is applied to prevent an overwhelming number of simple negative samples (e.g., background regions) from dominating the loss calculations. This increases the relative contribution of hard samples and prevents the model from converging to suboptimal local minima.
2. Emphasis on boundary negative samples (μ−0.1<x<μ): A progressively increasing exponential weight ${\;e}^{1-\mu }$ is assigned. Since *μ* ∈ (0,1), the weight remains consistently greater than 1, compelling the model to focus on difficult negative samples that have ambiguous classification. This reinforcement mechanism enhances the model’s robustness in detecting challenging scenarios such as occluded and small targets.
3. Decay weighting for positive samples (x≥μ): The weight, given by $e^{1-x}$, decays exponentially as the IoU increases. This ensures that hard positive samples (where *x*→$\mu^{+}$) receive weights similar to those of boundary negative samples, facilitating improved learning of ambiguous object features. In contrast, for easy positive samples (*x*→1), the weight asymptotically approaches 1, preventing high-confidence samples from disproportionately influencing the optimization process.

By integrating these adaptive weights into the binary cross-entropy (BCE) loss function, we derive the Slide Loss, which enhances model training by striking a balance between simple and hard samples. The formulation is expressed as follows:

(8) $$L_{Slide}=\frac{1}{N}\sum_{i=1}^{N}f\left(x_{i}\right)\cdot L_{BCE}\left(p_{i},\;y_{i}\right)$$

(9) $$L_{BCE}\left(p,\;y\right)=-\left[y\cdot {log}_{\sigma \left(p\right)}+\left(1-y\right)\cdot {log}_{\left(1-\sigma \left(p\right)\right)}\right]$$

where $f\left(x_{i}\right)$ is the weight assigned to the i-th sample, $p_{i}$ denotes its predicted score, and $y_{i}$ represents the corresponding ground-truth label. This loss function effectively mitigates the dominance of easy samples in training, ensuring that the model learns to recognize complex and ambiguous objects more effectively. The experimental results demonstrate that our method significantly improves both classification accuracy and localization robustness, making it a promising enhancement for object detection models in real-world scenarios.

## 4. Results

### 4.1. Experimental Environment and Parameter Settings

The experimental environment was Ubuntu 22.04, using an NVIDIA A10 GPU (CUDA 11.7.1) and an Intel Xeon Platinum 8369B CPU, and the model was built based on Python 3.9 and Torch 1.10. Taking YOLOv11n as the baseline, the improved model MAS-YOLOv11 was trained and tested in combination with the hyperparameter settings in Table 1 to verify its effectiveness.

### 4.2. Datasets

#### 4.2.1. DUO Dataset

**Figure 5:**
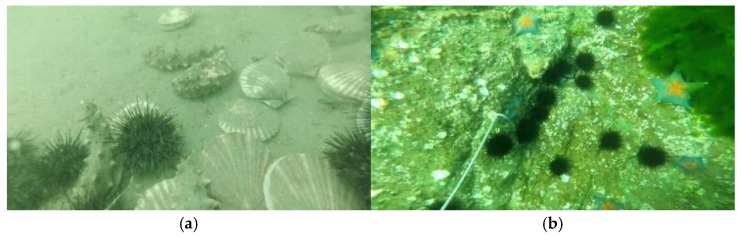
This is a sample image of the DUO dataset, in which (**a**) the image has degraded problems such as blurring and color cast, including holothurian, echinus, and scallop categories, and (**b**) the image has severe color cast, including starfish and echinus.

**Figure 6:**
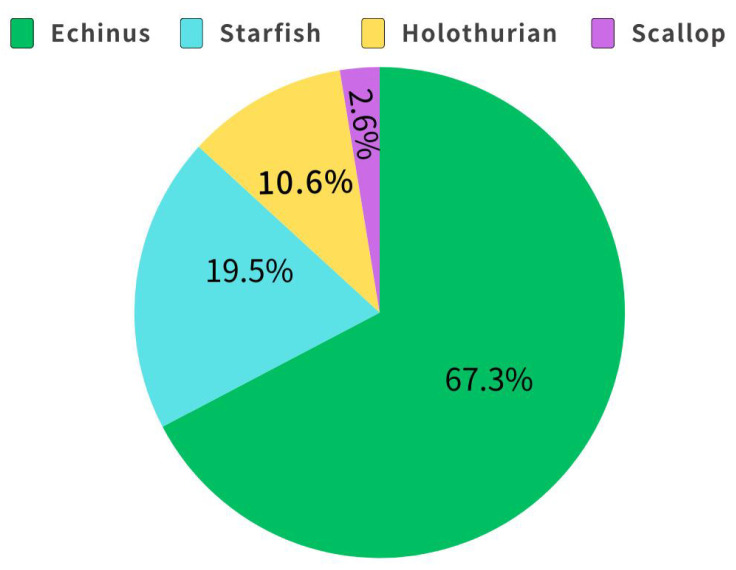
The distribution of the number of labeled objects by category in DUO.

The DUO dataset [36] integrates underwater images and precise annotations of the URPC series challenges. As shown in Figure 5, this dataset encompasses images at different depths with varying degrees of brightness, background, contrast, blurriness, and color deviation, truly reflecting the underwater target detection scene. The dataset contains a total of 7782 images, labeled with four types of targets: holothurian, echinus, scallop, and starfish, totaling 74,515 instances. As shown in Figure 6, the number of instances in each category shows a significant long-tailed distribution, and there is an unbalanced label distribution phenomenon, which is very likely to affect the model’s learning ability for difficult samples. In this paper, the training, validation, and test sets are divided at a ratio of 7:2:1 for evaluating the performance of the MAS-YOLOv11 model.

#### 4.2.2. RUOD Dataset

**Figure 7:**
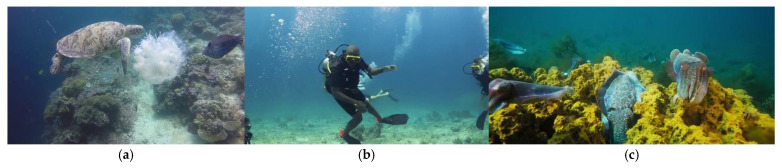
This is a sample of the RUOD dataset, where (**a**–**c**) are the categories of the redundant DUO dataset: (**a**) fish, turtles, and jellyfish; (**b**) divers; (**c**) corals and squid.

The RUOD [37] dataset is built on a real ocean observation platform, which highly restores diverse underwater scenarios and includes a variety of target types. The dataset has a total of 14,000 high-resolution images and 74,903 annotated objects, and the ratio of the training set to the validation set is 7:3. As shown in Figure 7, the RUOD dataset covers ten categories: holothurian, echinus, scallop, starfish, fish, corals, diver, cuttlefish, turtle, and jellyfish. At the same time, the dataset includes real complex underwater environments such as blur, color shift, and light interference, which can help effectively evaluate the adaptability, robustness, and accuracy of the object detection model in real underwater scenes.

### 4.3. Evaluation Metrics

To comprehensively evaluate the performance of the improved model, multiple evaluation metrics were employed, including precision (*P*), recall (*R*), F1-score, mean average precision (*mAP*), FPS (Frames Per Second), floating-point operations per second (GFLOPs), and the total number of model parameters. The mathematical formulations of these metrics are given as follows:

(10) $$P=\frac{TP}{TP+FP}$$

(11) $$R=\frac{TP}{TP+FN}$$

(12) $$F1=\frac{2*P*R}{P+R}$$

(13) $$AP=\int_{0}^{1}P\left(R\right)dR\;$$

(14) $$mAP=\frac{1}{N}\sum_{i=1}^{N}{AP}_{i}$$

where true positive (*TP*) represents the number of correctly identified positive samples, false positive (*FP*) denotes the number of negative samples that were incorrectly classified as positive, and false negative (*FN*) refers to the number of positive samples that were misclassified as negative.

Precision (P) measures the proportion of correctly predicted positive samples among all samples classified as positive by the model. It quantifies the reliability of the model’s positive predictions. Recall (R) indicates the proportion of actual positive samples that were correctly identified by the model. It reflects the model’s ability to detect positive instances. F1-score is the harmonic mean of precision and recall, providing a balanced measure of both metrics. Average precision (AP) evaluates the model’s performance for each individual class, integrating precision and recall across different confidence thresholds. Mean average precision (mAP) represents the mean AP across all object categories, serving as a comprehensive measure of the model’s overall detection accuracy. Here, *N* denotes the total number of object classes. FPS reflects the reasoning efficiency of the model and is an important indicator for evaluating real-time performance. Floating-point operations per second (GFLOPs) measures the computational complexity of the model by quantifying the total number of floating-point calculations performed during inference. Model parameters refer to the total number of learnable weights in the network, reflecting the model’s size and complexity. By jointly analyzing these metrics, a thorough assessment of the proposed model’s detection accuracy, computational efficiency, and generalization capability can be achieved.

### 4.4. Analysis of Experimental Results

#### 4.4.1. Performance Evaluation of the MAS-YOLOv11 Model

To comprehensively evaluate the effectiveness of the MAS-YOLOv11 model, a comparative analysis was conducted between the baseline model and the improved version. The corresponding results are presented in Table 2. Specifically, AP-Ho, AP-Ec, AP-Sc, and AP-St represent the average precision (AP) scores for detecting the holothurian, echinus, scallop, and starfish categories, respectively.

Compared to the baseline model, MAS-YOLOv11 achieves notable improvements in detection accuracy across all four categories, with AP increases of 6.1% for holothurian, 0.5% for echinus, 6.2% for scallop, and 0.7% for starfish. In addition to category-specific gains, MAS-YOLOv11 also demonstrates significant improvements across several key overall performance metrics: precision (P) increased by 1%, recall (R) by 3.7%, and the F1-score by 3%. Furthermore, the model shows an improvement of 3.5% in mAP@50 and 3.3% in mAP@50-95 compared to the baseline, confirming its enhanced detection capability.

**Figure 8:**
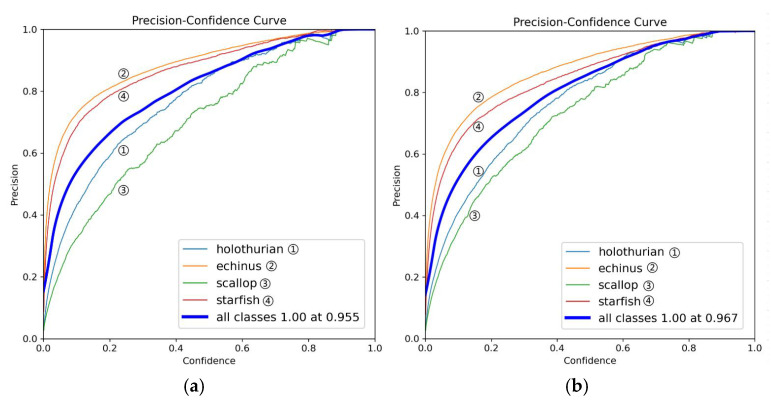
This is a precision comparison chart, where (**a**) is the YOLOv11 model and (**b**) is the MAS-YOLOv11 model.

As illustrated in Figure 8, the precision–confidence curves compare YOLOv11 (a) and MAS-YOLOv11 (b). Overall, MAS-YOLOv11 achieves higher precision across all categories, although the improvement is modest. Specifically, the echinus (orange curve) and starfish (red curve) categories maintain higher precision across the entire range of confidence thresholds, indicating enhanced feature extraction and more effective information learning. In the low-confidence range (confidence < 0.5), holothurian (blue) and scallop (green) show notable gains, reflecting improved detection of small or subtle targets.

**Figure 9:**
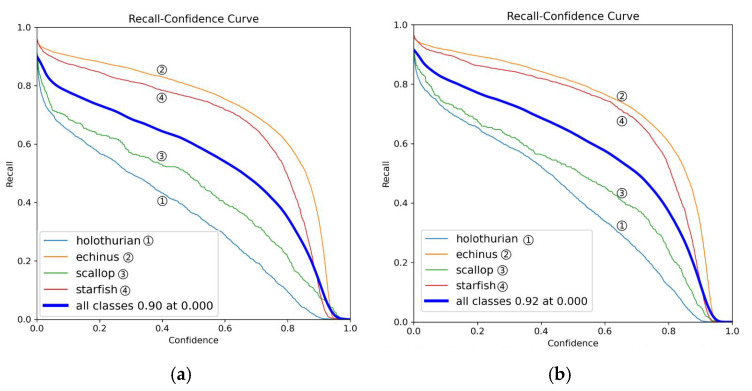
This is a recall comparison chart, where (**a**) is the YOLOv11 model and (**b**) is the MAS-YOLOv11 model.

As shown in Figure 9, the recall–confidence curves compare YOLOv11 (a) and MAS-YOLOv11 (b). At all confidence levels, MAS-YOLOv11 consistently achieves higher recall, indicating its ability to detect more true targets. Notably, when the confidence is below 0.5, recall improves significantly across all categories, demonstrating the model’s enhanced capability in detecting small, blurred, or occluded targets. Additionally, the smoother curve of MAS-YOLOv11 suggests improved feature extraction and target recognition, effectively reducing false and missed detections due to confidence fluctuations.

**Figure 10:**
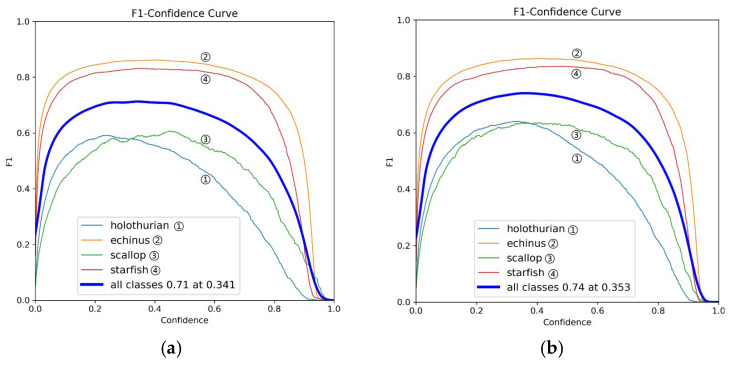
This is an F1 comparison chart, where (**a**) is the YOLOv11 model and (**b**) is the MAS-YOLOv11 model.

Figure 10 shows the F1-scores of YOLOv11 and MAS-YOLOv11 across different confidence thresholds. Compared with YOLOv11, MAS-YOLOv11 achieves a higher peak F1-score (0.74 vs. 0.71) and a higher optimal confidence threshold (0.353 vs. 0.341), reflecting an overall improvement in detection performance. Notably, MAS-YOLOv11 achieves consistently higher F1-scores for holothurians (blue curve), particularly at medium confidence levels (0.3–0.6), indicating a better ability to differentiate holothurians from the background, thus reducing both false positives and missed detections. Improvements are also observed for the scallop category (green curve), suggesting that MAS-YOLOv11 is more resilient to visual artifacts and underwater lighting interference. Furthermore, the smoother F1-score curve of MAS-YOLOv11 highlights its greater detection stability under varying confidence settings, effectively balancing precision and recall, and demonstrating its suitability for complex underwater object detection scenarios.

**Figure 11:**
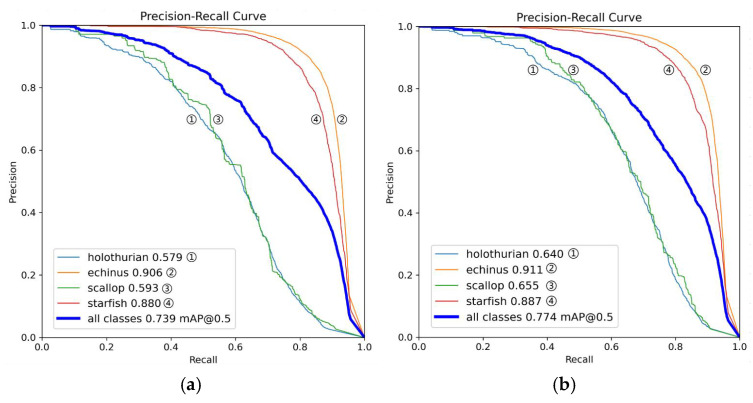
This is an mAP@50 comparison chart, where (**a**) is the YOLOv11 model and (**b**) is the MAS-YOLOv11 model.

Figure 11 shows the mAP@50 comparison graph. Compared to the baseline YOLOv11 (a), MAS-YOLOv11 (b) achieves improved accuracy across all categories, with particularly significant gains in detecting holothurians (blue) and scallops (green). This indicates that the model has a good false detection suppression effect on such targets. Overall, MAS-YOLOv11’s mAP@50 increased from 0.739 to 0.774—a 3.5% improvement—demonstrating its enhanced performance in small object detection and adaptability to complex backgrounds.

#### 4.4.2. Generalization Capacity Evaluation of MAS-YOLOv11

The RUOD dataset presents challenges such as texture degradation caused by image blurring, color cast, and insufficient illumination. Additionally, the target sizes vary widely, and their spatial distribution tends to be clustered, requiring the model to excel in both multi-scale detection and precise localization of dense targets. In contrast, the DUO dataset is characterized by severe color cast and image blurring, with a highly imbalanced distribution of target categories and significant size differences. It contains many small targets, which pose additional challenges for feature extraction.

To evaluate the generalization ability of MAS-YOLOv11, this study used identical training parameters and environmental settings for both the RUOD and DUO datasets, thereby eliminating experimental condition biases. The test results are summarized in Table 3 and Table 4, where Table 3 provides an overall performance evaluation of MAS-YOLOv11, while Table 4 details the detection accuracy for each category within the RUOD dataset.

As shown in Table 3, the MAS-YOLOv11 model achieved a precision (P) of 79.2%, a recall (R) of 69%, an F1-score of 73%, mAP@50 of 76%, and mAP@50-95 of 50.9% on the RUOD dataset. The differences between these metrics and those on the DUO dataset are all within 1.4%, indicating strong generalization performance. However, the relatively low mAP@50-95 suggests that there is room for improvement in localization accuracy under stricter IoU thresholds, particularly for small targets.

**Figure 12:**
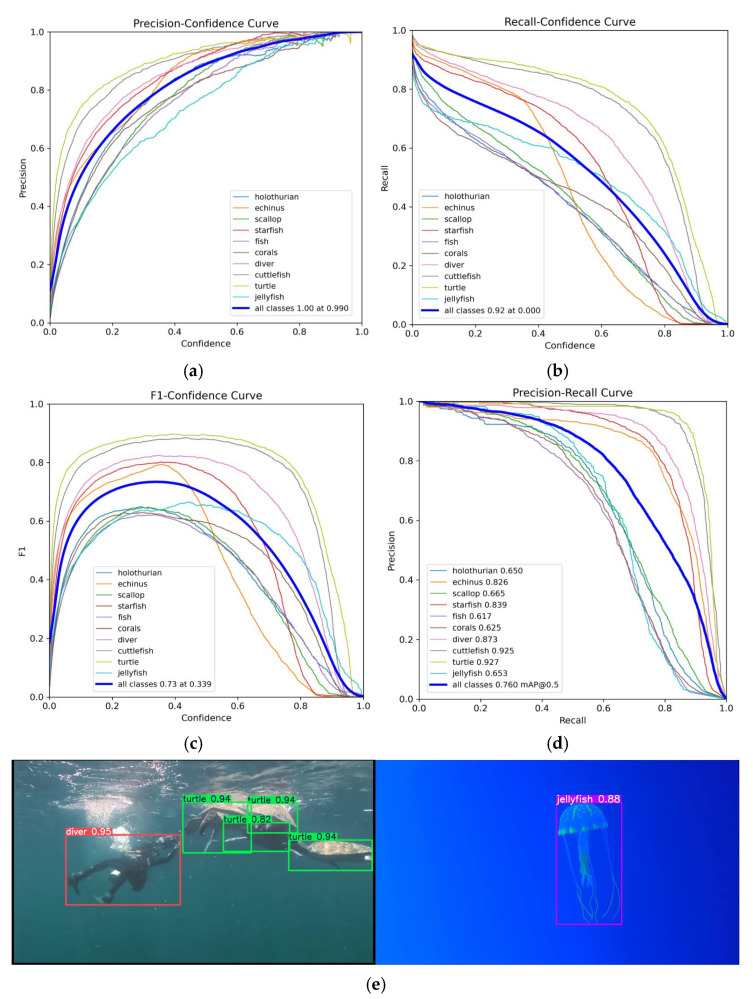
This is a RUOD result chart, where (**a**) is a precision plot, (**b**) is a recall plot, (**c**) is an F1 plot, (**d**) is an mAP@50 plot, and (**e**) is an example of a test results plot.

Table 4 shows that categories in less challenging areas, such as echinus, starfish, and divers, achieved detection accuracies above 80% on RUOD. Notably, squids and sea turtles exceeded 92%, highlighting the model’s strong ability to recognize targets with clear textures. For categories affected by complex backgrounds and dense distributions, such as holothurians, corals, and scallops, the model still maintains an average accuracy of 64.2%, demonstrating good generalization. To present these results more clearly and intuitively, the experimental data are visualized in Figure 12.

#### 4.4.3. Ablation Study

Ablation experiments are conducted to systematically analyze the impact of individual components in a model by employing a controlled experimental design. In this study, ablation experiments were performed on the DUO dataset to rigorously evaluate the effectiveness of the proposed improvement strategies in the MAS-YOLOv11 model. The quantitative results of these experiments are summarized in Table 5.

As shown in Table 5, the ASFFHead module effectively enhances the model’s performance, increasing precision (P) by 2.2%, recall (R) by 1.5%, and mAP@50 and mAP@50-95 by 2.5% and 2.7%, respectively. This indicates an overall optimization in multi-scale object detection. Additionally, the introduction of the Slide Loss function improves the classification loss, boosting recall by 1.6%. This helps address data imbalance and strengthens the model’s ability to learn from difficult samples. When all three improved modules are combined, recall and mAP@50 reach their highest values compared to the baseline, increasing by 3.7% and 3.5%, respectively. The Slide Loss function not only optimizes feature learning but also enhances processing speed when used alongside the C2PSA_MSDA and ASFFHead modules.

Although introducing the C2PSA_MSDA module alone into the backbone network results in limited improvements—recall (R) increases by only 0.8% and mAP@50 by 1.1%—its combination with the ASFFHead module demonstrates a clear synergistic effect. The experimental results show that this combined model achieves notable gains across key metrics: precision (P) rises by 1.7%, recall by 3.2%, and mAP@50 and mAP@50-95 by 3.2% and 3.6%, respectively.

**Figure 13:**
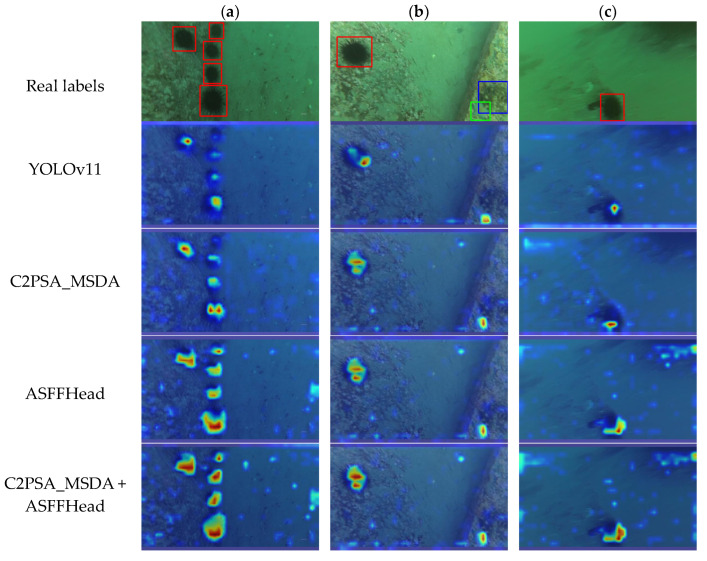
This is the Grad-CAM heatmap of MAS-YOLOv11, where (**a**) is a dense target with occlusion, (**b**) is a sparsely distributed multi-category target, and (**c**) is a blurred target.

As illustrated in Figure 13, when detecting occluded sea cucumbers, the combined model exhibits stronger activation of edge textures compared to both the baseline and single-module models. This indicates that the joint design overcomes the performance limits of individual modules by more effectively integrating multi-scale information, thereby enhancing the overall detection performance.

However, these improvements come with trade-offs. The combination increases the model’s parameter count by 1.38 M and reduces the processing speed by 37.4 FPS. This is mainly due to the overhead of real-time kernel generation in dynamic convolution, which involves layered cross-weight calculations.

#### 4.4.4. Comparative Experiments on Attention Mechanisms

To validate the architectural effectiveness of MAS-YOLOv11 and the synergy between the C2PSA_MSDA and ASFFHead modules, this study followed the experimental design outlined in Table 6. Various attention mechanisms—such as DAT [38], EMA [39], iEMA, and MLCA [40]—were integrated into the C2PSA module to create corresponding improved variants, which were then combined with ASFFHead for systematic comparative experiments. Notably, iEMA leverages the inverted residual structure from iRMB [41] to further optimize gradient propagation and reduce computational redundancy, while preserving the multi-branch cross-channel interaction characteristic of EMA. All experiments were conducted on the DUO dataset under consistent training parameters and environmental settings.

The results are summarized in Table 7. Compared to the control group, precision showed only a slight improvement with the EMA mechanism, a slight decrease with the MSDA and DAT mechanisms, and a significant decline with the other two attention mechanisms. Regarding recall (R), the MSDA mechanism demonstrated a notable increase of 1.7%, benefiting from the multi-scale perception advantage of dynamic convolution. DAT and EMA followed, with modest gains of 0.6% and 0.2%, respectively, while the remaining mechanisms saw significant decreases.

Considering the comprehensive metrics mAP@50 and mAP@50-95, only EMA and MSDA achieved positive improvements in both, with MSDA showing a more substantial gain (mAP@50: +0.7%, mAP@50-95: +0.9%), confirming the synergy between C2PSA_MSDA and ASFFHead. Specifically, C2PSA_MSDA enhances multi-scale context modeling through dynamic convolution, and when combined with ASFFHead’s feature-adaptive weighting, it improves the target localization and classification accuracy in complex scenes.

**Figure 14:**
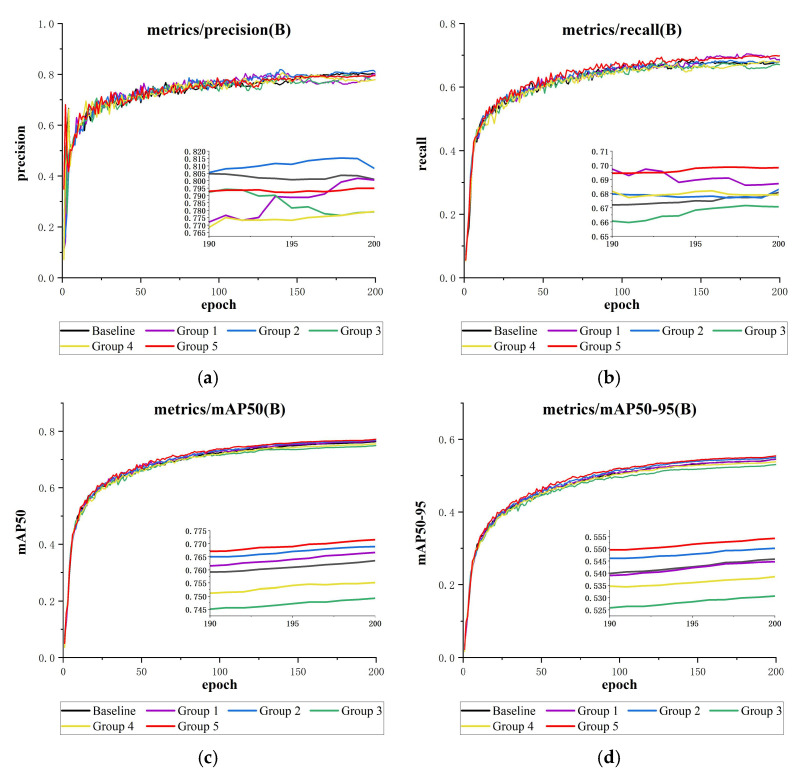
This is a comparison of the attention mechanisms’ results charts (containing detailed subgraphs for 190–200 epochs): (**a**) precision comparison, (**b**) recall comparison, (**c**) mAP@50 comparison, and (**d**) mAP@50-95 comparison.

However, incorporating the MSDA mechanism increases the model parameters by 0.02 M and reduces the inference speed by 7.6 FPS. This is mainly due to the computational overhead of dynamic convolution and the complexity of cross-scale feature interactions within MSDA. Figure 14 presents a graphical comparison of these evaluation metrics.

#### 4.4.5. Comparative Experiment with Loss Function

The YOLOv11 model employs location loss (DFL + CIoU) and classification loss (BCE). Given the characteristics of the DUO dataset, such as dense small targets and unbalanced distribution of sample categories, we designed four sets of comparative experiments based on the original loss function, as shown in Table 8: Groups 1–2 optimize the localization loss, and Groups 3–4 optimize the classification loss to verify the effectiveness of the Slide Loss.

**Figure 15:**
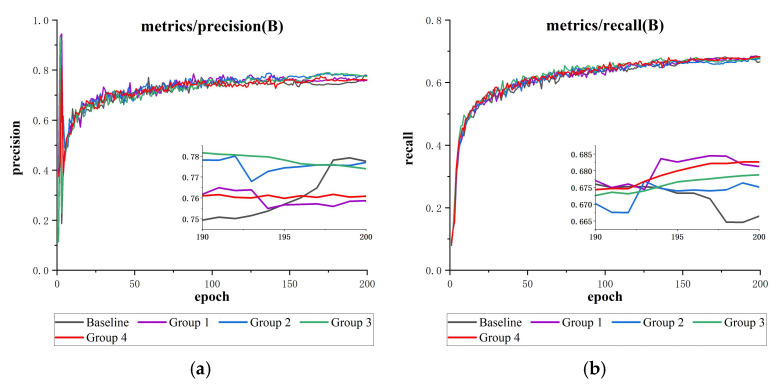

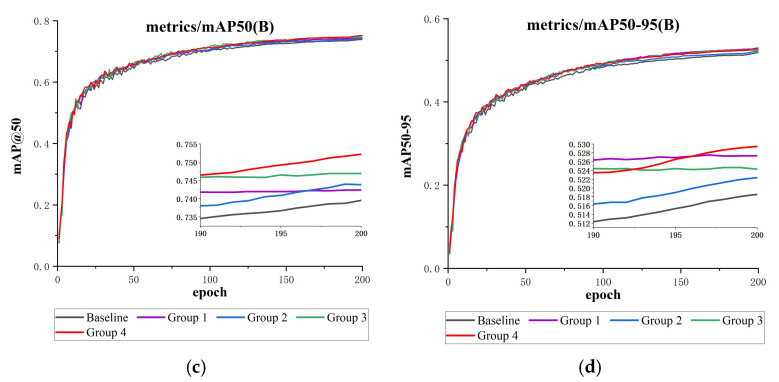
This is a loss function comparison results plot (containing detailed subgraphs for 190–200 epochs): (**a**) precision comparison, (**b**) recall comparison, (**c**) mAP@50 comparison, and (**d**) mAP@50-95 comparison.

As shown in Table 9, compared to the control group, Group 1 enhances the directional perception by introducing SIoU [42], and Group 2 improves the shape adaptability with ShapeIoU [43]. Both groups achieved modest recall (R) increases of 0.6% and 0.8%, respectively; however, precision (P) showed little improvement, likely due to the ambiguity of underwater target boundaries. Correspondingly, mAP@50-95 increased only slightly, by 0.4% to 0.7%. Group 3 applies Focaler Loss [44] to emphasize difficult samples, resulting in a recall increase of 1.2%. Group 4 introduces Slide Loss into the classification loss, dynamically adjusting the sample attention via sliding weights, and achieving a recall improvement of 1.8%, outperforming the other groups significantly. Comprehensive metric analysis showed that Slide Loss increased mAP@50 and mAP@50-95 by 1.3% and 1.1%, respectively, confirming its effectiveness in mitigating class imbalance through the dynamic allocation of sliding weights (see Figure 15).

#### 4.4.6. Model Comparison Experiments

**Figure 16:**
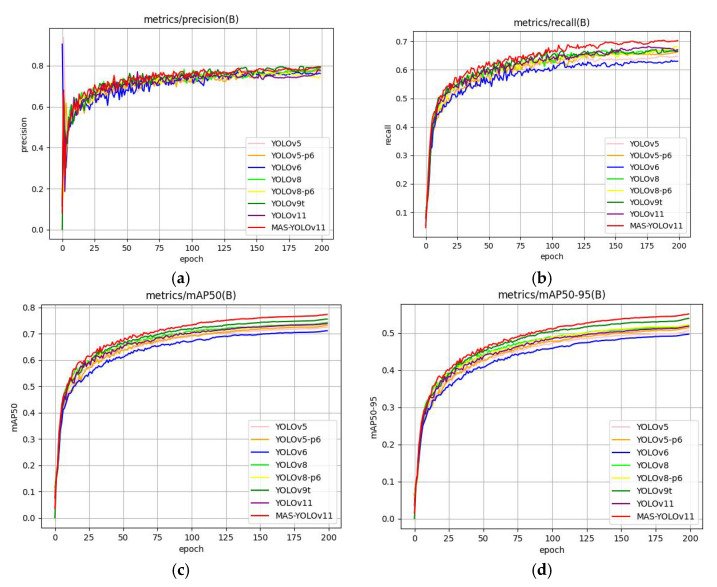
This is a diagram of a model comparison experimental results chart: (**a**) precision comparison, (**b**) recall comparison, (**c**) mAP@50 comparison, and (**d**) mAP@50-95 comparison.

To comprehensively evaluate the superiority of the proposed MAS-YOLOv11 model, we conducted a comparative analysis against several state-of-the-art object detection frameworks, including Faster R-CNN, YOLOv5 [45], YOLOv5-P6, YOLOv6 [46], YOLOv8 [47], YOLOv8-P6, and YOLOv9t [48]. All models were trained and evaluated on the DUO dataset under identical experimental conditions to ensure a fair performance comparison. The detailed experimental results are presented in Table 10. To display the experimental results more intuitively, the experimental data were visualized as shown in Figure 16.

Table 10 shows that MAS-YOLOv11 improves the classification accuracy by 1% compared to the baseline model, highlighting its enhanced ability to capture subtle differences in complex targets and improve category discrimination through an optimized feature learning strategy. Additionally, the model achieved a peak recall rate of 70.3% on the DUO dataset—an increase of 3.7% over the baseline—demonstrating a significant reduction in missed detections. This advantage is especially pronounced in low-visibility or occluded scenarios, where MAS-YOLOv11 effectively detects more true targets.

In terms of overall object detection performance, MAS-YOLOv11 achieves a 3.5% relative increase in mAP@50, reflecting a significant enhancement in detection capability and more balanced accuracy across multiple object categories. Under the more stringent mAP@50-95 metric, MAS-YOLOv11 surpasses all comparison models, reaching a peak score of 55.1%, which is 3.3% higher than that of YOLOv11. This result demonstrates the model’s good performance across varying intersection-over-union thresholds and its adaptability to diverse detection challenges in complex environments.

However, the improved detection accuracy comes with increased computational demands. Compared to YOLOv11, MAS-YOLOv11 has 23% more parameters and incurs a 43% higher computational load, resulting in a frame-rate drop from 109.3 FPS to 74.4 FPS.

This precision-for-resource trade-off means that MAS-YOLOv11 requires substantial computational resources, making it more suitable for environments where sufficient processing power is available to support its deployment.

**Figure 17:**
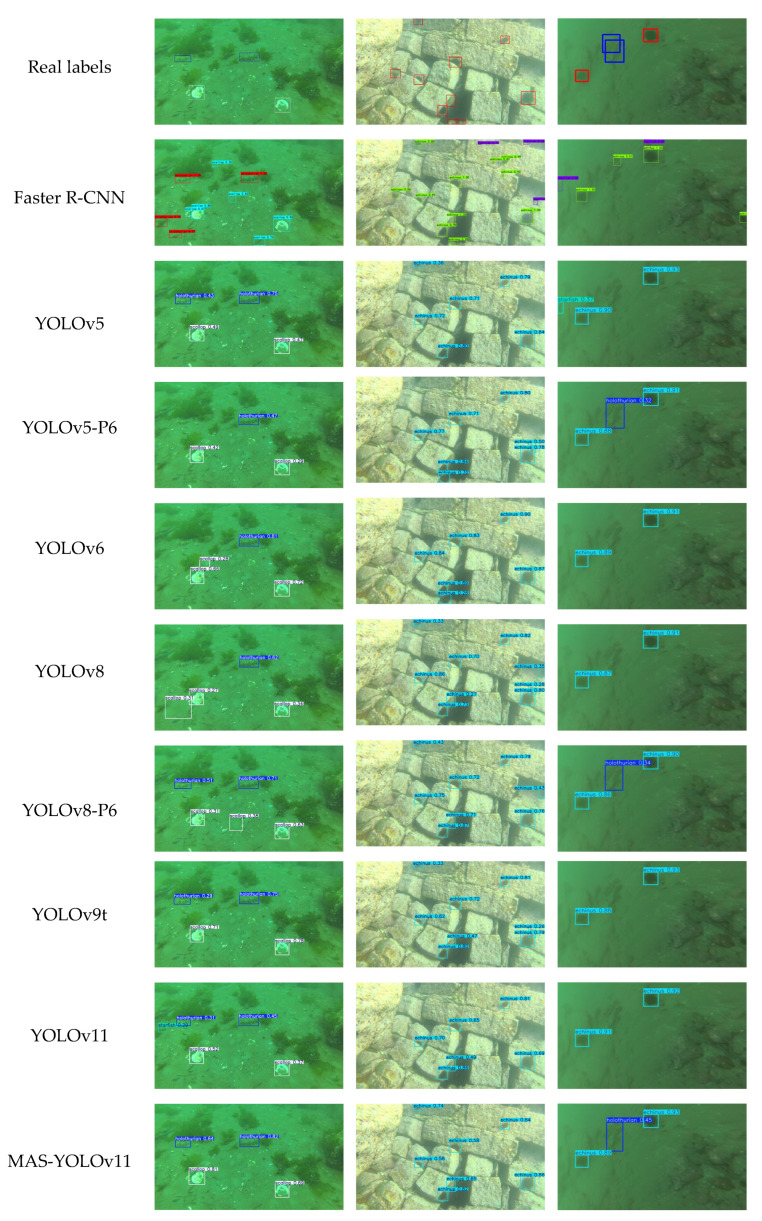
This is a comparison of the detection results of each model.

To further evaluate the performance of the proposed model, three representative images were selected to visually illustrate the detection results, as shown in Figure 17.

In the first scenario, characterized by limited sample size and partial occlusion, targets such as scallops and holothurians, both of which suffer from data scarcity, were missed or incorrectly detected by most of the comparison models. In contrast, MAS-YOLOv11 achieved complete and accurate detection. This result demonstrates that the integration of attention mechanisms and a feature enhancement strategy enables the model to extract salient texture information from a limited number of samples, effectively mitigating the impact of long-tailed data distributions and preserving target feature continuity under occlusion.

In the second scenario, involving densely distributed multi-target scenes, MAS-YOLOv11 exhibited clear advantages, achieving the lowest missed detection rate and eliminating false detections entirely. These improvements can be attributed to its enhanced cross-scale feature fusion mechanism, which effectively disentangles spatial relationships among closely packed objects. Additionally, the incorporation of a dynamic sampling strategy strengthens the model’s perceptual capability in occluded regions, further improving its detection robustness in complex environments.

The third scenario focuses on the detection of slender holothurian targets, which are typically difficult to identify due to their narrow shape and low visual saliency. Among all evaluated models, only MAS-YOLOv11, YOLOv5-P6, and YOLOv8-P6 successfully detected these targets, with MAS-YOLOv11 demonstrating the most consistent and precise performance. These findings further validate this model’s superior learning capability for challenging samples. By accurately capturing and enhancing discriminative features while suppressing irrelevant background information, MAS-YOLOv11 effectively reduces the likelihood of missed detections in complex underwater scenes.

#### 4.4.7. Model Robustness Testing

To verify the robustness of the MAS-YOLOv11 model, we systematically introduced Poisson noise and Gaussian noise of varying intensities to the DUO test set for simulation experiments.

Poisson noise was added to simulate the image degradation typical of underwater scenes, allowing for the assessment of the model’s anti-interference capability. This noise arises from the random nature of photon arrivals and varies with the signal intensity, exhibiting non-stationarity.

The experimental results are presented in Table 11. As the noise intensity increases, all performance metrics of the model gradually decline. Poisson noise notably increases the pixel-level uncertainty in low-illumination regions, causing blurred target boundaries. Under noise-free conditions, the model achieves mAP@50 and mAP@50-95 scores of 77.4% and 55.1%, respectively. At natural Poisson noise levels (scale = 1.0), mAP@50 drops to 50.2%, mAP@50-95 falls to 32.8%, and recall (R) decreases significantly to 44.3%. Under extreme noise conditions (scale = 0.1), mAP@50 plummets to 17.7%, nearly losing detection capability. These results indicate that while the model’s performance degrades substantially under strong Poisson noise, it still maintains a reasonable level of robustness at noise intensities below the medium range.

The equipment aging scenario was simulated by adding Gaussian noise with varying variance (σ). Gaussian noise, a stationary noise following a normal distribution, commonly occurs in sensors and signal transmission channels. As shown in Table 12, as σ increases from 0 to 20, the model’s recall rate sharply declines from 70.3% to 21.4%, while mAP@50 and mAP@50-95 drop from 77.4% and 55.1% to 25.7% and 16.1%, respectively. Gaussian noise degrades the target’s contour features, leading to severe missed detections. Notably, when σ ≥ 10, the detection performance deteriorates rapidly, highlighting the model’s sensitivity to medium- and high-intensity Gaussian noise.

Overall, MAS-YOLOv11 maintains good detection performance and demonstrates certain robustness under mild noise interference. However, its performance degrades significantly in high-noise environments, particularly with Gaussian noise. The complex underwater conditions often obscure key target features, complicating feature extraction and causing semantic ambiguity. Therefore, when deploying the model in real-world underwater scenarios, it is essential to incorporate image preprocessing techniques, such as adaptive denoising and specialized optimization strategies targeting noise sensitivity, to enhance the model’s robustness.

**Figure 18:**
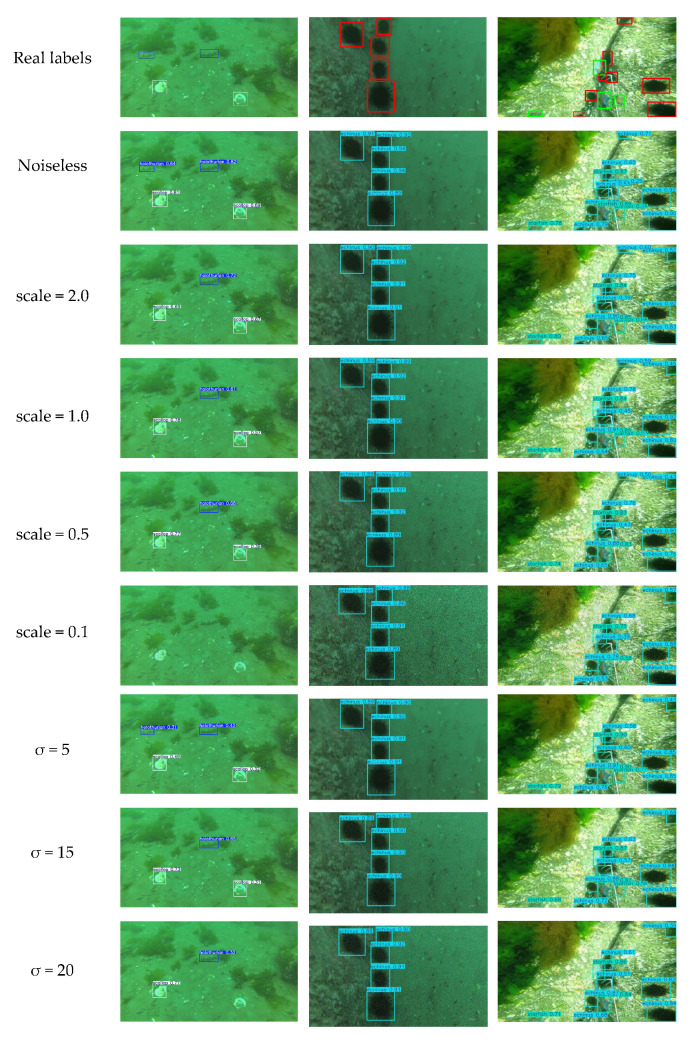
Test results at different noise intensities.

Figure 18 illustrates the object detection performance under varying noise intensities, where “scale” denotes the Poisson noise level and “σ” represents the Gaussian noise variance. As the noise intensity increases, the images progressively exhibit blurring and texture degradation, which severely impair the model’s ability to recognize targets. Consequently, missed detections become more frequent, and the detection accuracy declines steadily.

## 5. Discussion

### 5.1. Findings

The proposed MAS-YOLOv11 model introduces a series of innovative enhancements to the YOLOv11 framework, significantly improving its performance in underwater target detection. By integrating multi-scale dilated convolution and an attention mechanism, the C2PSA_MSDA module effectively captures long-range contextual information while preserving feature map resolution, which is particularly beneficial for handling the scale diversity of underwater objects.

Furthermore, the adaptive feature fusion strategy in the ASFFHead module addresses the limitations of traditional fixed-weight fusion mechanisms in Feature Pyramid Networks (FPNs). Its learnable spatial attention weighting mechanism dynamically suppresses cross-scale feature conflicts, ensuring comprehensive information fusion across different feature levels.

Additionally, Slide Loss, with its dynamic weighting strategy, enables the model to effectively mine hard samples under imbalanced dataset conditions, further refining its overall detection accuracy and robustness.

The experimental results demonstrate that MAS-YOLOv11 achieves significant performance improvements on the DUO dataset, with a recall rate increase of 3.7%, an F1-score improvement of 3%, and mean average precision (mAP) scores of 77.4% (mAP@50) and 55.1% (mAP@50-95), surpassing the baseline model by 3.5% and 3.3%, respectively. Furthermore, the model’s mAP@50 on the RUOD dataset reaches 76%, validating its generalization capability in different underwater environments.

### 5.2. Limitations and Future Works

Despite the promising detection performance achieved by MAS-YOLOv11, several limitations and challenges remain, particularly in the context of cross-domain generalization and real-time applicability in complex underwater environments.

In the generalization evaluation, MAS-YOLOv11 achieved a competitive mAP@50 of 76% on the RUOD dataset, which is comparable to its performance on the DUO dataset (77.4%). However, this seemingly marginal performance gap reveals the persistent domain adaptation problem in underwater object detection. Variations in underwater environments—such as differences in lighting, water turbidity, and object appearance—introduce significant domain shifts that hinder model robustness and transferability. Therefore, future experiments should incorporate domain adaptation evaluation metrics to more systematically assess the model’s cross-domain generalization capability.

To mitigate domain-induced performance degradation, future research should explore the integration of unsupervised domain adaptation (UDA) techniques, such as adversarial training or domain alignment strategies. Additionally, incorporating domain-aware modules and feature decoupling mechanisms may allow the model to learn more domain-invariant and semantically consistent representations, thereby enhancing its adaptability to unseen underwater scenarios. Knowledge distillation techniques could also be employed to transfer semantic knowledge from large-scale, well-annotated datasets to smaller, domain-specific datasets, helping the model to maintain its performance across diverse underwater conditions.

Although MAS-YOLOv11 has shown improved detection accuracy, this advancement comes at the cost of increased model complexity and a noticeable reduction in inference speed. This trade-off poses challenges for deployment in real-time applications, particularly in resource-constrained platforms such as underwater robotic systems. Therefore, future efforts should prioritize lightweight model design strategies, including dynamic sparse convolution, neural architecture search (NAS), pruning, and quantization. These techniques can effectively balance accuracy and efficiency, ensuring practical feasibility in time-sensitive underwater operations.

Moreover, while the model exhibits partial robustness under extreme conditions, as validated through synthetic noise perturbation tests, more comprehensive experiments are required to evaluate its resilience against other typical underwater challenges. Scenarios such as target occlusion, low visibility, and high contrast variations have not yet been sufficiently addressed. The development of synthetic occlusion datasets and controlled low-contrast simulations can provide a more systematic and rigorous assessment of model robustness under real-world underwater constraints.

In conclusion, while MAS-YOLOv11 demonstrates potential for underwater object detection, addressing its current limitations through enhanced domain adaptation, lightweight design, and robust evaluation strategies will be crucial for its advancement toward practical, real-world deployment.

## Figures and Tables

**Table 1:** Experimental environment parameters table.

Training Parameters	Values
Learning rate	0.01
Momentum	0.937
Weight decay	0.0005
Batch size	16
Optimizer	SGD
Image size	640 × 640
Epoch	200
Patience	20

**Table 2:** Comparison of the results before and after the improvement of the YOLOv11 model (unit: %). Bold font indicates the best performance values under the same evaluation metric.

Net	AP-Ho	AP-Ec	AP-Sc	AP-St	P	R	F1	mAP@50	mAP@50-95
YOLOv11	57.9	90.6	59.3	88	77.8	66.6	71	73.9	51.8
MAS-YOLOv11	**64**	**91.1**	**65.5**	**88.7**	**78.8**	**70.3**	**74**	**77.4**	**55.1**

**Table 3:** Overall evaluation index of the MAS-YOLOv11 model in the RUOD dataset (unit: %).

Net	P	R	F1	mAP@50	mAP@50-95
MAS-YOLOv11	79.2	69	73	76	50.9

**Table 4:** The detection accuracy of each category of RUOD (unit: %). The categories in the table, from left to right, are holothurian, echinus, scallop, starfish, fish, corals, divers, squid, turtles, and jellyfish.

Net	AP-Ho	AP-Ec	AP-Sc	AP-St	AP-Fi	AP-Co	AP-Di	AP-Cu	AP-Tu	AP-Je
MAS-YOLOv11	65	82.6	66.5	83.9	61.7	62.5	87.3	92.5	92.7	65.3

**Table 5:** Ablation test results, where “√” indicates the module is included (unit: %). Bold font indicates the best performance values under the same evaluation metric.

Net	C2PSA_MSDA	ASFFHead	Slide Loss	P	R	mAP@50	mAP@50-95	Parameter/M	FPS
1				77.8	66.6	73.9	51.8	2.59	**109.3**
2	√			77.1	66.8	74	52.1	2.60	96
3		√		**80**	68.1	76.4	54.5	3.96	79.5
4			√	76	68.2	75.2	52.9	2.59	107.4
5	√	√		79.5	69.8	77.1	**55.4**	**2.59**	71.9
6	√	√	√	78.8	**70.3**	**77.4**	55.1	**2.59**	74.4

**Table 6:** Experimental setup for attention mechanism comparison.

Experimental Group	Combination of Modules	Improvement Points
Baseline	ASFFHead	Control group
Group 1	DAT + ASFFHead	Improve the adaptability of the model to the deformation target
Group 2	EMA + ASFFHead	Optimize cross-channel interaction and improve the utilization of multi-scale features.
Group 3	iEMA + ASFFHead	Enhance feature fusion efficiency and reduce computational redundancy
Group 4	MLCA + ASFFHead	Balance the computational cost with feature discrimination
Group 5	MSDA + ASFFHead	Combined with dynamic convolution, it strengthens the target perception of complex scenes.

**Table 7:** Comparative results of attention mechanism (unit: %). Bold font indicates the best performance values under the same evaluation metric.

Experimental Group	P	R	mAP@50	mAP@50-95	Parameter/M	FPS
Baseline	80	68.1	76.4	54.5	3.96	79.5
Group 1	79.5	68.9	76.7	54.5	3.99	76.2
Group 2	**80.8**	68.3	76.8	55	**3.91**	80.3
Group 3	77.9	67.1	74.9	53.1	3.93	78.3
Group 4	77.9	67.9	75.5	53.9	**3.91**	**81.2**
Group 5	79.5	**69.8**	**77.1**	**55.4**	3.98	71.9

**Table 8:** Experimental setup for loss function comparison.

Experimental Group	Classification Loss	Localization Loss	Improvement Points
Baseline	BCE	DFL + CIoU	Control group
Group 1	BCE	DFL + SIoU	Improved orientation sensitivity for regression boxes
Group 2	BCE	DFL + ShapeIoU	Introduced shape-adaptive weighting
Group 3	Focaler Loss	DFL + CIoU	Dynamically focus on difficult samples
Group 4	Slide Loss	DFL + CIoU	Slide to adjust the weights between samples

**Table 9:** Comparison of loss functions (unit: %). Bold font indicates the best performance values under the same evaluation metric.

Experimental Group	P	R	mAP@50	mAP@50-95
Baseline	**77.8**	66.6	73.9	51.8
Group 1	75.9	68.1	74.2	52.7
Group 2	77.7	67.5	74.4	52.2
Group 3	77.4	67.9	74.7	52.4
Group 4	76.1	**68.3**	**75.2**	**52.9**

**Table 10:** This is a model comparison results table (unit: %). Bold font indicates the best performance values under the same evaluation metric.

Net	P	R	mAP@50	mAP@50-95	Parameter/M	FLOPs/G	FPS
Faster R-CNN	53.7	56.4	62.9	37.3	60.13	82.7	37.2
YOLOv5	77.7	64.9	72.5	50.6	2.19	**5.9**	131.8
YOLOv5-P6	77.3	66.3	73.3	51.5	3.69	6.0	100.7
YOLOv6	76.5	62.9	71.2	49.7	4.16	11.6	**158.5**
YOLOv8	**79.4**	66.5	74.3	52.1	2.69	6.9	133.7
YOLOv8-P6	74.4	68.2	73.9	52.2	4.34	6.9	112.2
YOLOv9t	79.4	67.1	75.6	54	**1.77**	6.7	61.4
YOLOv11	77.8	66.6	73.9	51.8	2.59	6.4	109.3
MAS-YOLOv11	78.8	**70.3**	**77.4**	**55.1**	3.98	8.6	74.4

**Table 11:** Poisson noise test results (unit: %).

Scale Value	Noise Intensity	P	R	mAP@50	mAP@50-95
0	None	78.8	70.3	77.4	55.1
2.0	Extremely weak	73.5	56.3	63.2	42.2
1.5	Weak	72.3	51.4	58.2	38.7
1.0	Natural Poisson	68.0	44.3	50.2	32.8
0.5	Medium	59.2	31.6	35.7	23.0
0.1	Strong	56.3	11.3	17.7	10.7

**Table 12:** Gaussian noise test results (unit: %).

Scale Value	Noise Intensity	P	R	mAP@50	mAP@50-95
0	None	78.8	70.3	77.4	55.1
5	Extremely weak	77.1	65.3	72.8	50.3
10	Mild	71.5	46.6	52.8	34.7
15	Medium	60.2	31.4	35.7	22.8
20	Strong	52.5	21.4	25.7	16.1

## Data Availability

The data presented in this study are available upon request from the corresponding author. The code cannot be shared due to specific reasons.

## References

[1] Lin S.W. (2023). The Core Essence and Contemporary Value of Xi Jinping’s Maritime Power Strategy. Theory Horiz..

[2] Li B.Z., Hua M.M., Jiang X.X. (2022). Problems and Path Selection of Marine Ecological Environment Governance in China. China Water Transp..

[3] Yang B., Fu H., Guo S.Q., Wang J. (2023). Marine Ecological Protection and Green Development in China. Sci. Technol. Rev..

[4] Yu H. (2020). Research Progress on Aquatic Animal Target Detection and Tracking Technology and Applications. J. Dalian Ocean Univ..

[5] Zhang Y., Yang F., He W. (2020). An Approach for Underwater Image Enhancement Based on Color Correction and Dehazing. Int. J. Adv. Robot. Syst..

[6] Ding Z. (2024). Research on the Degradation Mechanism of Underwater Optical Imaging Quality. Ph.D. Thesis.

[7] Zhang Y.T., Huang D.Q., Wang D.W., He J.J. (2023). Review of Object Detection Algorithms Based on Deep Learning and Their Applications. Comput. Eng. Appl..

[8] Cheng E., Lin X., Chen Y., Yuan F., Yang W. (2016). GLCM-Based No-Reference Perceptual Blur Metric for Underwater Blur Images. Int. J. Circuits Syst. Signal Process..

[9] Yahya M.F., Arshad M.R. Robust Recognition of Targets for Underwater Docking of Autonomous Underwater Vehicle. Proceedings of the 2016 IEEE/OES Autonomous Underwater Vehicles (AUV).

[10] Susanto T., Mardiyanto R., Purwanto D. Development of Underwater Object Detection Method Based on Color Feature. Proceedings of the 2018 International Conference on Computer Engineering, Network and Intelligent Multimedia (CENIM).

[11] Redmon J., Divvala S., Girshick R., Farhadi A. You Only Look Once: Unified, Real-Time Object Detection. Proceedings of the 2016 IEEE Conference on Computer Vision and Pattern Recognition (CVPR).

[12] Liu W., Anguelov D., Erhan D., Szegedy C., Reed S., Fu C.Y., Berg A.C. (2016). SSD: Single Shot MultiBox Detector. Computer Vision—ECCV 2016, Proceedings of the 14th European Conference on Computer Vision, Amsterdam, The Netherlands, 8–16 October 2016.

[13] Girshick R., Donahue J., Darrell T. Rich Feature Hierarchies for Accurate Object Detection and Semantic Segmentation. Proceedings of the 2014 IEEE Conference on Computer Vision and Pattern Recognition (CVPR).

[14] Ren S., He K., Girshick R. (2017). Faster R-CNN: Towards Real-Time Object Detection with Region Proposal Networks. IEEE Trans. Pattern Anal. Mach. Intell..

[15] Liu Z., Du J., Tian F. (2019). MR-CNN: A Multi-Scale Region-Based Convolutional Neural Network for Small Traffic Sign Recognition. IEEE Access.

[16] Lin T.Y., Dollar P., Girshick R. Feature Pyramid Networks for Object Detection. Proceedings of the 2017 IEEE Conference on Computer Vision and Pattern Recognition (CVPR).

[17] Xin F., Zhang H., Pan H. (2023). Hybrid Dilated Multilayer Faster R-CNN for Object Detection. Vis. Computer..

[18] Ye Z.B., Duan X.H., Zhao C. (2023). Research on Improved YOLOv3-SPP for Underwater Target Detection. Comput. Eng. Appl..

[19] Guo L., Zhang Y., Tian Q., Ran Y. (2023). Underwater Fish Detection Based on ECA-YOLOv5. Comput. Syst. Appl..

[20] Liu P., Yang H.B., Song Y. (2020). Marine Biological Identification Algorithm Based on Improved YOLOv3 Network. Appl. Comput. Res..

[21] Chang J., Chen H.F., Wang B.B. (2024). Underwater Image Enhancement Based on Parallel Guidance of Transformer and CNN. Comput. Eng. Appl..

[22] Cao R., Zhang R., Yan X., Zhang J. (2024). BG-YOLO: A Bidirectional-Guided Method for Underwater Object Detection. Sensors.

[23] Zhang Y., Xingshan L.I., Yemei S.U.N., Shudong L.I.U. (2022). Underwater Object Detection Algorithm Based on Channel Attention and Feature Fusion. J. Northwest Polytech. Univ..

[24] Knausgård K.M., Wiklund A., Sørdalen T.K., Halvorsen K.T., Kleiven A.R., Jiao L., Goodwin M. (2022). Temperate Fish Detection and Classification: A Deep Learning-Based Approach. Appl. Intell..

[25] Chen X., Yuan M., Yang Q., Yao H., Wang H. (2023). Underwater YCC: Underwater Target Detection Optimization Algorithm Based on YOLOv7. J. Mar. Sci. Eng..

[26] Xin S.A., Ge H.B., Yuan H., Yang Y.D., Yao Y. (2024). Lightweight Underwater Target Detection Algorithm Based on Improved YOLOv7. Comput. Eng. Appl..

[27] Liu X.J., Liu Y., Jiang S.X. (2023). Underwater Target Detection Based on SimAM Attention Mechanism and DCN-YOLOv5. J. Chongqing Univ. Commer. (Nat. Sci.).

[28] Zhao H., Xu C., Chen J., Zhang Z., Wang X. (2025). BGLE-YOLO: A Lightweight Model for Underwater Bio-Detection. Sensors.

[29] Yi W.G., Yang J.W., Yan L.W. (2024). Research on Underwater Small Target Detection Technology Based on Single-Stage USSTD-YOLOv8n. IEEE Access.

[30] Jiao J., Xu C., Zhang J., Ma L., Bai Y., Yang W., Gao Y., Gao Y. (2023). DilateFormer: Multi-Scale Dilated Transformer for Visual Recognition. IEEE Trans. Multimed..

[31] Liu S., Huang D., Wang Y. (2019). Learning Spatial Fusion for Single-Shot Object Detection. arXiv.

[32] Yu Z., Huang H., Chen W., Su Y., Liu Y., Wang X. (2022). YOLO-FaceV2: A Scale and Occlusion Aware Face Detector. arXiv.

[33] Luo S., Li K.Y., Wu J.H., Ren P. (2024). Underwater Occluded Target Detection Algorithm Based on Adversarial Attention Mechanism. Comput. Eng. Appl..

[34] Zhang C., Chen X.P., Han G.Q., Liu X.J. (2023). Spatial Transformer Network on Skeleton-Based Gait Recognition. Expert Syst..

[35] Wen C., He W., Wu W., Liang X., Yang J., Nong H., Lan Z. (2024). Recognition of Mulberry Leaf Diseases Based on Multi-Scale Residual Network Fusion SENet. PLoS ONE.

[36] Liu C., Wang T., Zhang X., Wu J., Zheng J., Feng Y. (2021). A Dataset and Benchmark of Underwater Object Detection for Robot Picking. Proceedings of the IEEE International Conference on Multimedia & Expo Workshops (ICMEW).

[37] Fu C., Liu R., Fan X., Wu Y., Wang L. (2023). Rethinking General Underwater Object Detection: Datasets, Challenges, and Solutions. Neurocomputing.

[38] Xia Z., Pan X., Song S., Li L.E., Huang G. (2022). Vision Transformer with Deformable Attention. Proceedings of the IEEE/CVF Conference on Computer Vision and Pattern Recognition (CVPR).

[39] Ouyang D., He S., Zhan J., Guo H., Huang Z., Luo M.L., Zhang G.L. (2023). Efficient Multi-Scale Attention Module with Cross-Spatial Learning. Proceedings of the IEEE International Conference on Acoustics, Speech and Signal Processing (ICASSP).

[40] Wan D., Lu R., Shen S., Xu T., Lang X., Ren Z. (2023). Mixed Local Channel Attention for Object Detection. Eng. Appl. Artif. Intell..

[41] Zhang J., Li X., Li J., Liu L., Xue Z., Zhang B., Jiang Z., Huang T., Wang Y., Wang C. (2023). Rethinking Mobile Block for Efficient Attention-Based Models. Proceedings of the IEEE/CVF International Conference on Computer Vision (ICCV).

[42] Gevorgyan Z. (2022). SIoU Loss: More Powerful Learning for Bounding Box Regression. arXiv.

[43] Zhang H., Zhan S. (2023). Shape-IoU: More Accurate Metric Considering Bounding Box Shape and Scale. arXiv.

[44] Zhang H., Zhang S. (2024). Focaler-IoU: More Focused Intersection over Union Loss. arXiv.

[45] Ultralytics YOLOv5. https://github.com/ultralytics/yolov5.

[46] Li C., Li L., Jiang H., Weng K., Geng Y., Li L., Ke Z., Li Q., Cheng M., Nie W. (2022). YOLOv6: A Single-Stage Object Detection Framework for Industrial Applications. arXiv.

[47] Jocher G., Qiu J., Chaurasia A. (2023). Ultralytics YOLO (Version 8.0.0) [Computer Software]. https://github.com/ultralytics/ultralytics.

[48] Wang C.Y., Yeh I.H., Liao H.Y.M. (2024). YOLOv9: Learning What You Want to Learn Using Programmable Gradient Information. arXiv.

